# Musical Expertise Affects Audiovisual Speech Perception: Findings From Event-Related Potentials and Inter-trial Phase Coherence

**DOI:** 10.3389/fpsyg.2019.02562

**Published:** 2019-11-15

**Authors:** Marzieh Sorati, Dawn Marie Behne

**Affiliations:** Department of Psychology, Norwegian University of Science and Technology, Trondheim, Norway

**Keywords:** speech perception, prediction, audiovisual, musical training, event-related potential (ERP), inter-trial phase coherence (ITPC), musicians, non-musicians

## Abstract

In audiovisual speech perception, visual information from a talker's face during mouth articulation is available before the onset of the corresponding audio speech, and thereby allows the perceiver to use visual information to predict the upcoming audio. This prediction from phonetically congruent visual information modulates audiovisual speech perception and leads to a decrease in N1 and P2 amplitudes and latencies compared to the perception of audio speech alone. Whether audiovisual experience, such as with musical training, influences this prediction is unclear, but if so, may explain some of the variations observed in previous research. The current study addresses whether audiovisual speech perception is affected by musical training, first assessing N1 and P2 event-related potentials (ERPs) and in addition, inter-trial phase coherence (ITPC). Musicians and non-musicians are presented the syllable, /ba/ in audio only (AO), video only (VO), and audiovisual (AV) conditions. With the predictory effect of mouth movement isolated from the AV speech (AV−VO), results showed that, compared to audio speech, both groups have a lower N1 latency and P2 amplitude and latency. Moreover, they also showed lower ITPCs in the delta, theta, and beta bands in audiovisual speech perception. However, musicians showed significant suppression of N1 amplitude and desynchronization in the alpha band in audiovisual speech, not present for non-musicians. Collectively, the current findings indicate that early sensory processing can be modified by musical experience, which in turn can explain some of the variations in previous AV speech perception research.

## 1. Introduction

Perception is shaped by information coming to multiple sensory systems, such as information from hearing speech and seeing a talker's face coming through the auditory and visual pathways. Early studies Klucharev et al. (2003) and Van Wassenhove et al. (2005) showed that this audiovisual information facilitated perception. Further research added that the visual information from facial articulations, which begins before the sound onset, can also work as a visual cue that leads the perceiver to form some prediction about the upcoming speech sound. This prediction by phonetically congruent visual information can modulate early processing of the audio signal (Stekelenburg and Vroomen, 2007; Arnal et al., 2009; Pilling, 2009; Baart et al., 2014; Hsu et al., 2016; Paris et al., 2016a,b, 2017). Insight into the influence of multisensory experiences, such as musical training, is only beginning to unfold (Petrini et al., 2009a,b, 2011; Lee and Noppeney, 2011, 2014; Paraskevopoulos et al., 2012; Behne et al., 2013; Proverbio et al., 2016; Jicol et al., 2018), and how this regulates audiovisual modulation in speech is yet to be understood.

Behavioral research on audiovisual (AV) speech has shown that visual cues from mouth, jaw and lip movements that start before the onset of a corresponding audio signal can facilitate reaction time and intelligibility in speech perception, compared with perception of the corresponding audio only (AO) condition (Schwartz et al., 2004; Paris et al., 2013). Electrophysiological evidence also indicates that modulation due to visual speech cues prior to the auditory onset is accompanied by amplitude and latency reduction in auditory event-related potentials (ERPs), such as N1 (Paris et al., 2017), which is the negative deflection elicited approximately 100 ms after a sudden acoustic change in the environment and also sensitive to attention (Näätänen and Picton, 1987; Näätänen et al., 2011). Furthermore, visual speech congruent with the auditory signal can speed up and decrease the later component, P2 (Van Wassenhove et al., 2005), which is fronto-central distributed and evoked around 200 ms after the audio onset (Pratt, 2014).

The N1/P2 waveform is an auditory ERP response which is generally related to physical attributes of an auditory stimulus, such as speech (Näätänen and Winkler, 1999; Tremblay et al., 2006), and both N1 and P2 are sensitive to previous experiences, such as musical training (Shahin et al., 2003). However, N1 and P2 have different scalp distributions and have different temporally and spatially underlying processes (Huhn et al., 2009); whereas the medial territory of Heschl's gyrus constitutes one of the primary sources of the N1 component, P2 responses are strongly dependent on the recruitment of auditory association cortex (Bosnyak et al., 2004; Kühnis et al., 2014).

Functional magnetic resonance imaging (fMRI) studies in AV speech perception have shown that auditory and visual sensory-specific pathway projections extend to the multisensory cortical regions, such as superior temporal sulcus (STS) in AV speech processing (Calvert, 2001; Sekiyama et al., 2003; Kreifelts et al., 2007). A combined fMRI and magnetoencephalography (MEG) study (Arnal et al., 2009) suggested that AV speech perception involves two functionally distinct pathways with two different time courses. An early feed-forward cortical pathway routes from the motion-sensitive cortex in the visual area to the auditory cortex. AV modulation at N1 due to the visual cue before the sound onset is processed through this early feed-forward pathway. In addition, this pathway is sensitive to general attributes of the stimuli, such as visual predictability (Arnal et al., 2009; Paris et al., 2017), temporal features (Senkowski et al., 2007; Pilling, 2009; Vroomen and Stekelenburg, 2010; Paris et al., 2017), and spatial location (Stekelenburg and Vroomen, 2012). Moreover, a later feedback projection through STS reflects the AV congruency between the visual information and perceived sound, which modulates P2 (Van Wassenhove et al., 2005; Arnal et al., 2009; Paris et al., 2016b).

A meta-analysis (Baart, 2016) of twenty different experiments with AV /ba/ showed that AV modulation does not always lead to N1 amplitude reduction. While some studies (e.g., Stekelenburg and Vroomen, 2007) suggested that the visual information predicting the upcoming sound might suppress the AV modulation at N1, others do not show N1 amplitude suppression in AV perception (Paris et al., 2016b). From these findings on visual speech inducing N1 amplitude suppression, two general considerations arise: the experimental task and participants' characteristics related to AV experience.

Variability across studies in the meta-analysis (Baart, 2016) may be dependent on factors such as experimental task and design (Luck, 2014; Baart, 2016). For example, N1 is sensitive to inverse modifications from attention and prediction: whereas auditory N1 is enhanced in response to attended stimuli, predictable stimuli often suppress N1 (Paris et al., 2016a). In AV speech perception, in which visual cues predict the up-coming sound while the participant is attending the stimulus, modification due to attention and prediction are confounded depending on the experiment design. Therefore, the direction of N1 amplitude (suppression or enhancement) may depend on different factors in the experiment that contribute to orientation of attention and/or predictability of the stimulus (Lange, 2013).

In the meta-analysis (Baart, 2016), selection criteria for participants in experiments were mainly based on age, auditory and vision tests (Stekelenburg and Vroomen, 2007; Pilling, 2009; Paris et al., 2016a). Some experiments (Besle et al., 2004; Van Wassenhove et al., 2005; Paris et al., 2016b) also controlled for participants' previous AV experience, such as native language, which can influence AV speech perception (Chen and Hazan, 2007; Wang et al., 2009, for review see, Heald et al., 2017). However, none of the studies in the meta-analysis reported the participants' musical experience, even though studies comparing musicians and non-musicians have suggested that previous musical training may shape AV perception (Musacchia et al., 2007; Lee and Noppeney, 2011; Paraskevopoulos et al., 2012; Proverbio et al., 2016). The current study, extends this previous research and controls for the musical background of participants by comparing musicians and non-musicians with the purpose of investigating the role of musical experience when visual cues predict the upcoming audio signal in AV speech perception.

Musical experience provides an attractive model for studying experience-based neural plasticity. Years of musical practice such as playing an instrument can enhance auditory processing (Zatorre et al., 2007; Strait and Kraus, 2014) and practicing a musical instrument offers a rich multimodal experience, integrating different sensory signals, including audio and visual information (Petrini et al., 2009a,b, 2011; Lee and Noppeney, 2011, 2014; Behne et al., 2013; Jicol et al., 2018). For example, a behavioral study by Petrini et al. (2009a) showed that drummers, compared to non-musicians, were more sensitive to AV synchronicity for drumming point-light displays and can even perceptually replace missing visual information (Petrini et al., 2009b). In a following fMRI study with similar stimuli (Petrini et al., 2011), they also showed that drummers had decreased neural activities compared to non-musicians. These studies indicate that previous AV experiences, such as musical experience, can shape music perception.

Extensive musical experience enhances auditory perception related to sub/cortical processing, not only in response to music, such as pitch perception (Kishon-Rabin et al., 2001; Schön et al., 2004; Zatorre et al., 2007; Bianchi et al., 2017) but also transferring beyond music to speech (e.g., Musacchia et al., 2008; Lima and Castro, 2011; Patel, 2011; Lee and Noppeney, 2014). Playing a musical instrument is an AV experience and growing evidence illustrates that musical expertise can benefit the encoding of other AV events such as speech due to the anatomical overlap in the brain circuitries involved in music and speech (Patel and Iversen, 2007; Kraus and Chandrasekaran, 2010; Patel, 2011; Shahin, 2011; Jantzen et al., 2014) both cortically (Shahin et al., 2003; Bidelman et al., 2014) and subcortically (Musacchia et al., 2007; Wong et al., 2007; Parbery-Clark et al., 2009). For instance, Musacchia et al. (2008) showed that musicians had an enhanced N1-P2 complex in response to an audio speech syllable compared to non-musicians and that this enhancement correlates with the subcortical enhancement of speech in musicians. These findings showed that N1 and P2 are prominent components modified by musical experience.

While N1 and P2 amplitudes and latencies can provide insights into the neural basis of musical experience and AV modulation based on the time-domain, the generation of evoked potentials such as N1 and P2 are also dependent on superposition of the trial-by-trial phase alignment of low-frequency (<30 Hz) EEG oscillations in response to a stimulus (Gruber et al., 2004; Eggermont, 2007; Edwards et al., 2009; Koerner and Zhang, 2015; van Diepen and Mazaheri, 2018). A combination of ITPC and ERP have previously been used to study early auditory ERP components both for adults (Koerner and Zhang, 2015) and children (Yu et al., 2018) and shown that ITPC data in delta, theta and alpha might be a predictor for early auditory ERP components such as N1 and P2. With this basis, in the current study phase-locking neural synchrony will be computed as inter-trial phase coherence (ITPC) to examine the role of each frequency band, which coincides with early auditory ERP components, including delta (1–4 Hz), theta (4–8 Hz), alpha (8–12 Hz), and beta (12–30 Hz) (Edwards et al., 2009). Smaller ITPC values indicate poorer consistency in the phase synchronicity of oscillations across trials and higher ITPC values indicate higher synchronicity across trials (Cohen, 2014).

Previous research on AV perception showed that visual cues predicting the upcoming syllable might result in resetting the phase of the ongoing oscillatory activity (Lakatos et al., 2007; Busch and VanRullen, 2010). Such oscillatory effects are involved in the processing of the cross-modal prediction, which is also associated with early evoked potentials (Arnal and Giraud, 2012). For example, low-frequency power, such as theta activity, which has been related to syllable encoding of speech (Giraud and Poeppel, 2012; Doelling et al., 2014) is suppressed in response to AV speech (Lange et al., 2013). Theta ITPC also significantly correlates with early ERP components (Koerner and Zhang, 2015). Correlated with later ERP components, theta oscillatory activity together with delta activity signals further processing of correctly predicted stimuli (Arnal et al., 2011). When visual prediction is congruent with the auditory signal, delta phase-locking activity increases while theta phase-locking activity decreases (Arnal et al., 2011, for review see, Arnal and Giraud, 2012). Moreover, suppression of EEG oscillations in alpha and beta frequency bands has been associated with prediction during sensory processing (Todorovic et al., 2015; Gisladottir et al., 2018) and when attention is oriented toward an upcoming stimulus (van Ede et al., 2014). Furthermore, alpha activity suppressed for AV speech perception (Lange et al., 2013) may also be related to the suppression mechanism during selective attention toward the anticipatory upcoming stimuli (for review see, Foxe and Snyder, 2011).

Other research suggests that musical experience modulates oscillatory networks, and compared to non-musicians, musicians showed higher ITPC values. For example, Doelling and Poeppel (2015) have shown that musical experience modified cortical entrainment, mainly in delta and theta band activities, which also affected the perceptual accuracy for musicians. Furthermore, studies have shown that musical experience shaped oscillatory networks such as alpha and beta activities in response to both speech (Bidelman et al., 2014; Bidelman, 2017), and non-speech stimuli (Trainor et al., 2009). In AV speech perception, visual cues predicting the upcoming sound are expected to decrease amplitudes and latencies for N1 and P2 components. With N1 and P2 coinciding with ITPC in low-frequency bands (Edwards et al., 2009), ITPC is also expected to show smaller values in the AV condition compared to the auditory condition. Therefore, the role of musical experience in AV speech perception will be investigated for ITPC in the delta, theta, alpha, and beta frequency bands.

With this basis, the current study has been designed, first, to replicate previous findings in auditory speech perception for musicians and non-musicians. As found in previous research (e.g., Musacchia et al., 2008), in audio speech perception musicians are expected to have enhanced N1 and P2 amplitudes compared to non-musicians. Moreover, previous research (e.g., Stekelenburg and Vroomen, 2007) suggested that phonetically congruent visual cues predicting an upcoming audio signal modulate AV speech by reducing N1 and P2 amplitudes and latencies. However, a meta-analysis (Baart, 2016) showed that N1 and P2 results have variations across studies. In the current study, participants' musical background will be controlled as musical background is a factor which can shape AV perception (Musacchia et al., 2008) and create variation across studies. Musicians and non-musicians' N1 and P2 amplitudes and latencies will be compared in audio and AV speech in an additive model to examine whether N1 and P2 amplitudes and latencies are reduced in AV speech compared to the audio speech. Furthermore, the current study also extends previous research by investigating whether AV modulation of speech is modified by previous AV experience, such as playing a musical instrument. Finally, as ITPC for lower frequencies, such as theta, are coincident with N1 and P2 (Edwards et al., 2009), ITPC is expected to show the same pattern as N1 and P2 and decrease in AV speech compared to audio speech. To examine if musicians show a general ITPC enhancement relative to non-musicians, group ITPC differences are assessed for audiovisual modulation in the delta, theta, alpha and beta bands.

## 2. Materials and Methods

Data in the current experiment were recorded as part of a larger project on AV perception. Here, only the method related to the speech stimuli will be reported.

### 2.1. Design

The current experiment investigated the effect of musical experience on audio and AV speech perception by comparing musicians and non-musicians' EEG in response to audio, video, and audio video conditions. First, to replicate previous studies, musicians and non-musicians were compared for N1 and P2 in response to audio syllables. Then, building on previous research, AV modulation in speech perception was examined for musicians and non-musicians by comparing auditory and audiovisual speech for N1 and P2 amplitudes and latencies, as well as inter-trial phase coherence (ITPC).

### 2.2. Participants

As summarized in Table 1, participants were 41 young adults, aged 19–33 years, of which 18 were musicians (9 female, mean age = 23 years, *SD* = 3 years) and 21 were non-musicians (10 female, mean age = 23 years, *SD* = 3 years). Data from two musicians were excluded due to technical issues. All participants were right-handed based on a variant of the Edinburgh Handedness Inventory (Oldfield, 1971), had normal-to-corrected visual acuity (Snellen test), and normal hearing (pure tone audiometry threshold of 15 dB HL or better for 250–4,000 Hz, British Society of Audiology, 2004). All participants had Norwegian as a first language, and none of the participants reported a history of neurological disorders. All of the participants provided written consent consistent with the Norwegian Center for Research Data (NSD) and were given an *honorarium* after the experiment.

**Table 1:** Means and standard deviations (in parentheses), for musicians and non-musicians based on a questionnaire.

	**Age**	**Gender**	**Interest in music (1= “not interesting at all” 10 = “very interesting”)**	**Listening to music per week**	**Age of starting an instrument**	**Musical experience**
Musicians	23 years (3 years)	9 females, 9 males	9 (1)/10	19 hr (13 hr)	8 years (2 years)	14 years (3 years)
Non-musicians	23 years (3 years)	10 females, 11 males	5 (2)/10	5 hr (5 hr)	-	Less than a year

Musicians were current students at the Norwegian University of Science and Technology (NTNU) and had Music Performance Studies or Musicology as their field of study. Admission for these programs requires passing theoretical and practical musical evaluations, in addition to demonstrating advanced instrumental skills. All were playing a musical instrument actively (average practice per week = 15 h, *SD* = 10 h) and regularly performed publicly during the experiment's timeframe. Musicians started formal music training at a mean age of 8 years (SD = 2 years) and had been playing their main instrument for at least 8 years (average years of playing the instrument = 14 years, SD = 3 years). All musicians had piano or keyboard as their main or secondary instrument, as well as expertise in at least one other instrument (e.g., guitar, percussion). Although some studies (Pantev et al., 2001; Strait et al., 2012) reported the instrument-specific effects of musical experience, more recent studies (e.g., Kühnis et al., 2013) suggested that the variation in musical instruments is not expected to affect the results since the general effects of musical training as an AV experience, rather than instrument-specific processes, modify AV perception. Therefore, for the current study, musicians were recruited based on their previous musical experience rather than their expertise with a specific musical instrument.

The musicians' self-reported interest in music was on average 9 out of 10 (1 = “not interesting at all” and 10 = “very interesting”). To isolate the effect of musical training to instrumentalists, musicians with dancing and vocal training were not included in this study (Hänggi et al., 2010; Halwani et al., 2011). None of the musicians reported having absolute pitch perception.

The non-musicians were also registered students at NTNU, although none were students of music. They had no more music training than the one year of weekly music training mandatory in Norwegian elementary schools. Their self-reported interest in music was on average 5 out of 10.

### 2.3. Stimuli

**Figure 1:**
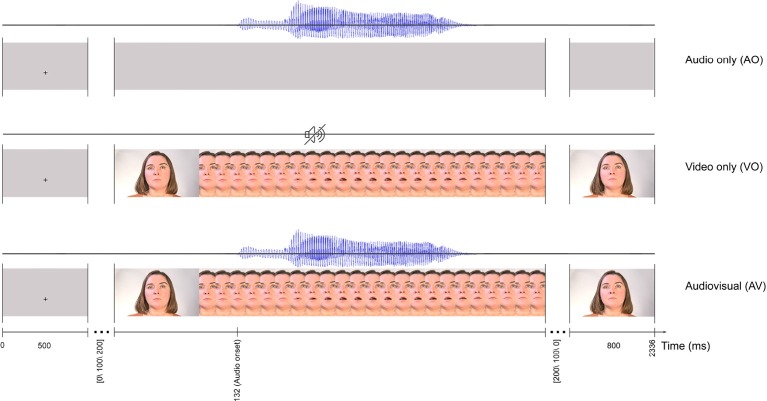
The trial timeline for three conditions: audio only (AO), video only (VO), and audiovisual (AV). All three conditions start with a 500 ms fixation cross and finish with a 800 ms still image as the last frame. Each frame in the figure represents two frames in the actual stimuli.

Stimuli were based on AV recordings of the syllable /ba/ spoken at an average fundamental frequency of 205 Hz by an adult female native speaker of Norwegian with a Trønder accent. As shown in Figure 1, the video in the recording was of her face while articulating the syllable. AV materials were recorded in an IAC sound-attenuated studio (IAC acoustics, Hampshire, UK) in the Department of Psychology's Speech Laboratory at NTNU. For the recordings, a Sony PMW-EX1R camera (30 fps) was connected to an external Røde NT1-A microphone (Sydney, Australia), mounted on a tripod.

The syllable /ba/ begins with a consonant that is formed at the lips, which being highly visible, conveys more predictive visual information than, for example, to syllables formed at the back of the mouth (e.g., Arnal et al., 2011). Among numerous productions of the syllable /ba/, one recording was chosen based on clear articulation of the syllable.

Speech processing is influenced by variation in acoustic attributes of different speakers' voices and variation across different tokens by the same speaker (e.g., Zhang, 2018). In the current study, the use of an AV recording from one speaker is motivated by the study's focus on AV, rather than auditory, perception. In the current AV study, acoustics details are isolated by directly comparing AV−VO with the same speaker's AO. Therefore, if musicians and non-musicians differ in their ERP or ITPC data, the difference would be independent of the acoustic attributes of the speaker's voice.

The AV recording of the selected syllable /ba/ was edited in Adobe Premiere Pro CS54.5, with videos exported in H.264 format with MP4 container, to develop three sets of /ba/ stimuli: audio only (AO), video only (VO) and, audio video (AV). In the AO condition, the 530 ms audio signal was presented with a gray visual background. In the VO condition, the original video recording from the speaker was presented with no audio. In the AV condition, the original AV recording of /ba/ was used (Figure 1).

### 2.4. Procedure

The experiment took place in an IAC sound-attenuated, dimly lit, studio at the Speech Laboratory, NTNU.

A participant's head was positioned on a chinrest throughout the experiment to minimize movement and maintain a stable head position relative to the display. The use of a chinrest also ensured that the participant's vision was directed to the monitor. The visual stimuli were presented on a 40″ LCD flat panel display (Samsung SyncMaster 400DX-2) with a resolution of 1,152 × 648, positioned at eye level, 190 cm in front of the participants. The video size was chosen so that the speaker's head size in the video was similar to the actual size of the speaker's head. Auditory stimuli were presented binaurally via ER1-14B insert earphones with HB7 Headphone buffer (Tucker-Davis Technologies, US). The audio stimuli were adjusted to an average sound pressure level of 65 dB.

For the three sets of AV stimuli (AO, VO, and AV), the audio and video delays for presenting stimuli on the monitor and through the earphones were recorded with an audiovisual delay test toolbox (Electrical Geodesics, Oregon, US) together with the EEG system (Electrical Geodesics, Oregon, US). The delays of 57 ms (±2 ms jitter) for video and 50 ms (±12 ms jitter) for audio were compensated later in the analysis.

The experiment was a sensory level target detection task with target trials which were used to ensure that participants were engaged in the task (9% of the trials) (adapted from Stekelenburg and Vroomen, 2007). Targets were the same modality as non-target trials, as research shows that attention modulates activity in the sensory cortices corresponding to the modality of the stimulus (Wild et al., 2012). Specifically, target trials in the AO condition included a 120 ms tone occurring 200 ms after the stimuli onset, in the VO trials a 120 ms-white dot occurred above or below the mouth and in the AV condition a synchronized tone with a white dot occurred. Target trials were excluded from the analysis.

Prior to the experiment, participants were instructed to limit eye movements, as well as to remain focused and yet stay relaxed during the experiment. They were also instructed on how to perform the experimental task and detect the target trials by pushing a button on a Response Pad 200 (Electrical Geodesics, USA). After receiving the instructions, the participant was presented a set of 5 practice trials to make sure that she/he learned the experimental task.

In the experiment, 327 trials were presented in each of three blocks (AO, VO, AV) for a total, of 981 pseudo-randomized trials.

As illustrated in Figure 1, each trial started with a 500 ms fixation cross against a gray background at the location on the monitor where the lips would be in the video, and this constituted the inter-stimulus interval. To avoid stimulus presentation phase-locked alpha activity for participants (Woodman, 2010; Luck, 2014), the fixation cross was followed by a still face image with a random interval of [0,100, 200] ms until the video started. Consistent with the speaker's natural lip and jaw movements when uttering the syllable, the video onset (the first detectable lip movement frame) was 132 ms preceding the auditory onset. Each stimulus lasted for a total of 1,536 ms (42 frames), and the last frame of the video was displayed for 800 ms.

The experiment took about an hour with 3-min breaks between blocks and short pauses in each block.

### 2.5. EEG Recordings

EEG data was recorded at 1,000 samples per second with a 128-channel dense array EEG system, with a Net Amps 300 amplifier (Electrical Geodesics, Oregon, US). Psychtoolbox (Pelli and Vision, 1997) together with Net Station (5.2.0.2) was used to present stimuli and record the responses. An independent online display was used for the experimenter to observe stimulus presentation and participant responses during the experiment. No online filters were applied, and Cz was the reference. Prior to EEG recording, participant head size was measured based on the nasion-inion and the left-to-right preauricular distance to select the best fit from the adult cap sizes, and the cap was placed with Cz at the midpoint of the nasion. Impedances were kept below 100 *KΩ*.

### 2.6. Data Analysis

#### 2.6.1. Pre-processing

Raw EEG recordings were interpolated to the 10–20 system (Jasper, 1958) and imported into Matlab R2015b. EEGLAB (v15) extension (Delorme and Makeig, 2004) and custom Matlab scripts were used for the entire analysis. Due to the slow direct current (DC) drifts in raw data, a 0.5 Hz (12 dB/octave) high-pass filter was applied to avoid displacement of peak amplitudes (Cohen, 2014). Then, a low-pass filter (48 Hz, 12 dB/octave) was applied, bad channels were removed, and the remaining channels were re-referenced offline to the average reference. Based on visual inspection, large artifacts, such as movements and large muscular artifacts, were removed from the recordings. Independent component analysis (ICA) was then applied to remove stereotypical eye blinks.

#### 2.6.2. Event-Related Potential (ERP)

EEG recordings were segmented into 800 ms epochs, starting 200 ms before and ending 600 ms after audio stimulus onsets. Baseline correction was performed from −200 ms to 0 ms. In the current study neural activity is recorded on different trials than behavioral responses were logged and epochs with participant responses (i.e., targets) were therefore excluded from further analysis.

N1 was scored in a window of 70–150 ms, and P2 was scored in a window of 120–250 ms (Stekelenburg and Vroomen, 2007, 2012; Paris et al., 2016b, 2017). As both N1 and P2 had a central maximum, Cz was chosen for calculating peak amplitudes and latencies for N1 and P2. In a 10–20 system, Cz is a derivative of waveforms from nearby electrodes among the original 128 channels, reflects the activities originating from auditory-related brain regions (Bosnyak et al., 2004), and has been broadly used in previous research in the field (e.g., Baart, 2016). Separately for musicians and non-musicians, average ERPs for each condition were calculated based only on the non-target trials. Therefore, in the current study neural activity is recorded on different trials than behavioral responses were logged.

As it has been suggested that musicians have enhanced N1 and P2 amplitudes (Musacchia et al., 2008), the first analysis focused on the difference between musicians and non-musicians at N1 and P2 in the AO condition.

To determine the role of visual cues predicting the upcoming auditory signal in AV compared to the auditory condition, previous literature has taken two approaches: A+V ≠ AV (e.g., Van Wassenhove et al., 2005; Brandwein et al., 2010) and AV−VO ≠ AO (e.g., Baart, 2016, a meta-analysis with twenty different experiments; Stekelenburg and Vroomen, 2012; Paris et al., 2016a, 2017, with non-speech stimuli). Here, the AV−VO ≠ AO model has been used to allow a comparison between the current results and those summarized in the recent meta-analysis (Baart, 2016), where only studies using the AV−VO ≠ AO model were included. Therefore, for further analyses, VO waveforms were subtracted from AV waveforms (AV−VO) to remove the contribution of the visual signal from the ERPs. First, to assess the amplitude and latency reduction at N1 and P2 due to the visual cues predicting the upcoming audio signal (Stekelenburg and Vroomen, 2007), N1 and P2 from the AV−VO were compared to N1 and P2 from the AO condition for each group. Furthermore, to examine the difference between musicians and non-musicians, the two groups were compared based on their N1 and P2 amplitudes and latencies in AO vs. AV−VO.

Finally, to explore the spatio-temporal dynamics of AO and AV interactions, pointwise two-tailed *t*-tests were conducted for AO and AV−VO at C3 and C4 in a 1–250 ms window, corresponding to N1 and P2. The differences between C3 and C4 for each group were considered significant when at least 12 consecutive points (12 ms while the signal sampling rate is 1,000 Hz) were significantly different (Stekelenburg and Vroomen, 2007). These analyses also allowed for detection of the earliest time point at which a potential difference in C3 and C4 occurred in AO and AV−VO.

#### 2.6.3. Inter-trial Phase Coherence (ITPC)

EEG recordings were segmented into 2,400 ms epochs starting 1,200 ms before and ending 1,200 ms after audio stimulus onsets. To calculate ITPC in delta (0.5–4 Hz), theta (4–8 Hz), alpha (8–12 Hz), and beta (12–30 Hz) with the “newtimef” function from the EEGLAB package (Delorme and Makeig, 2004), frequencies between 0.5 Hz and 48 Hz were decomposed, beginning with 1 Morlet wavelet cycle while linearly increasing cycles. The results are 194 complex ITPC values at a constant frequency step (0.2461 Hz). Further, the complex ITPC values averaged in each frequency bands (delta, theta, alpha and beta) and then, the magnitude of averaged complex values has been calculated. The maximum of ITPC values within the designated time window of 72–225 ms, corresponding to N1 and P2 components were identified for each participant for further analysis.

ITPC represents an estimate of the phase synchrony across the EEG trials as a function of the time point and frequency in the epoch time series:

(1) $$\begin{array}{l} ITPC_{tf}=|n^{-1}\overset{n}{\underset{r=1}{\sum }}e^{ik_{tfr}}| \end{array}$$

In Equation (1), t stands for time, f for frequency, n for the number of trials, and *eik* index is the Fourier transform component at time t and frequency f.

ITPC reflects the amount of phase synchronization at each time-frequency point. ITPC results bound between zero and one, with zero indicating completely randomly distributed phase angles and one indicating completely identical phase angles (Cohen, 2014).

Currently, reporting the number of trials to measure the strength of ITPC (Cohen, 2014) analysis is not common practice. To address this issue, ITPC as a function of the number of randomly selected trials was calculated and compared to the critical *p*-value threshold of 0.01 for each frequency band, to show how many trials are sufficient to have statistically significant results.

#### 2.6.4. Statistical Analysis

An analysis of variance (ANOVA, α = 0.05), was conducted (SPSS, v. 25) to examine the statistical significance for the repeated measures background factor (musicians vs. non-musicians) in the AO condition. For further analyses, a two-way ANOVA was conducted to assess the interaction between the experimental condition (AO vs. AV−VO) and background (musicians vs. non-musicians) on N1 and P2 latencies and amplitudes at electrode Cz. In the main effect of background, data from AO and AV−VO are collapsed, which would not give a meaningful comparison between the two groups. A separate analysis comparing musicians and non-musicians in AO perception and the interaction between background and condition would be more precise. Therefore, the main effect of background is not part of the hypotheses in this study and not directly addressed below but is reported in **Table 4**. An ANOVA was also conducted for examining inter-trial phase locking in the delta, theta, alpha, and beta frequency bands.

For a reliable statistical analysis with EEG, factors that affect the signal-to-noise ratio like the number of trials for each condition and noise, are determinative. For example, assuming that the noise in the system and environment is minimum, it is suggested to have a fixed set of trials for specific components to have a reliable result (Luck, 2014). For ITPC analyses, calculating the strength of ITPC is possible based on the number of trials (Cohen, 2014). To evaluate the strength of ITPC, a bootstrapping algorithm was run between 75 and 225 ms for each frequency band. First, a Gaussian function centered at the middle of each frequency band (center frequency for delta (2.5 Hz), theta (6 Hz), alpha (10 Hz), and beta (21 Hz) was used as a wavelet function to run the convolution over the signal. The bootstrap algorithm with 50 iterations was run for each trial. The bootstrap algorithm returns the average ITPC for the selected time window and the convoluted signal. Results were then evaluated for statistical significance (*p* < 0.01). This process was repeated for the AO and AV−VO conditions for musicians and non-musicians. To determine the required number of trials for all conditions and groups, the maximum number of needed trials among all conditions and groups were treated as the minimum threshold (*n* = 980).

## 3. Results

Musicians detected 96% and non-musicians 95% of the target trials (Table 2), with high response percentages and low standard deviations for musicians and non-musicians indicating that both groups of participants attentively focused on the stimuli during the experiment. Both groups showed slightly fewer correct responses for the VO condition, which might be due to blinking at the same time as the 120 ms-white target dot occurred.

**Table 2:** Musicians and non-musicians' correct responses in percentage and standard deviations in parenthesis, in response to the target in AO, VO, and AV trials.

	**Audio only condition**	**Video only condition**	**Audio visual condition**	**Average**
Musicians	98% (0)	92% (1)	99% (1)	96% (2)
Non-musicians	96% (1)	92% (1)	97% (1)	95% (3)

### 3.1. Event-Related Potential (ERP)

**Figure 2:**
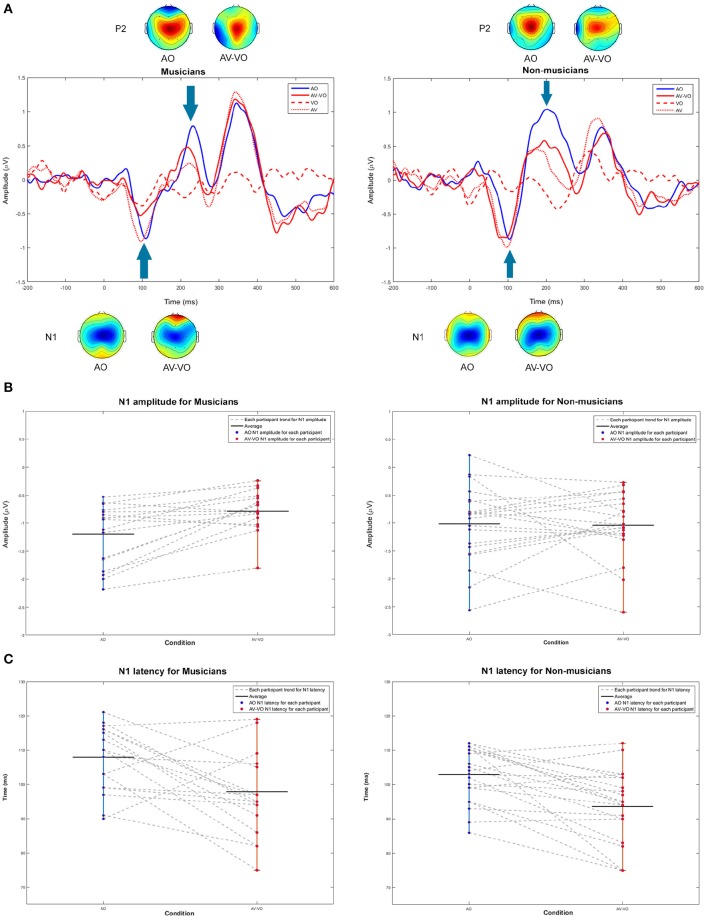
**(A)** Grand averaged waveforms at Cz and topographical maps in N1 and P2 windows for audio only (AO) in blue, video only (VO) in red dashed-line, audiovisual (AV) in red dotted-line, and audiovisual minus video only (AV−VO) in red line. **(B,C)** Shift functions at N1 amplitudes and latencies for musicians and non-musicians. Each gray dashed-line connect each participants AO and AV−VO data points.

Figure 2 shows ERP waveforms at Cz for AO and AV−VO for musicians and non-musicians. First, musicians and non-musicians were compared based on their N1 and P2 amplitudes and latencies in AO condition. Then, the effect of adding visual cues to the AO condition (AV−VO vs. AO) was examined and compared between the two groups to assess the effect of previous musical experience in response to AV speech with predictive visual cues preceding the upcoming sound.

#### 3.1.1. Audio-Only Condition

Musicians and non-musicians were compared for the AO condition. A one-way analysis of variance (ANOVA) was carried out for AO N1 amplitude [*F*_(1, 37)_ = 0.80, *p* = 0.37] and AO N1 latency [*F*_(1, 37)_ = 3.21, *p* = 0.08]. Although the average AO N1 latency for musicians was 5 ms later than non-musicians (Table 3), neither N1 amplitude nor N1 latency showed a significant group difference.

**Table 3:** Mean and standard deviation (SD) of N1 and P2 amplitude (μ*V*), latency (ms) and ITPC for delta, theta, alpha, and beta activity, for musicians and non-musicians.

		**Event-related potential (ERP)**				**Inter-trial phase coherence (ITPC)**			
		**N1**		**P2**					
		**Amplitude (μV)**	**Latency (ms)**	**Amplitude (μV)**	**Latency (ms)**	**Delta**	**Theta**	**Alpha**	**Beta**
Musicians	AO	−1.19 (0.53)	108 (10)	1.33 (0.53)	222 (31)	0.23 (0.09)	0.24 (0.07)	0.16 (0.05)	0.12 (0.03)
	AV−VO	−0.78 (0.36)	98 (13)	0.85 (0.4)	206 (28)	0.21 (0.08)	0.21 (0.08)	0.09 (0.05)	0.11 (0.02)
Non-musicians	AO	−1.01 (0.67)	103 (8)	1.65 (1.02)	213 (31)	0.25 (0.09)	0.25 (0.1)	0.18 (0.08)	0.15 (0.05)
	AV−VO	−1.04 (0.56)	93 (11)	1.06 (0.81)	209 (27)	0.21 (0.09)	0.22 (0.07)	0.17 (0.04)	0.11 (0.03)

To further investigate the differences between the two groups associated with AO perception, AO P2 amplitude and latency were submitted to a one-way ANOVA to compare musicians and non-musicians. Both AO P2 latency [*F*_(1, 37)_ = 0.93, *p* = 0.34] and AO P2 amplitude [*F*_(1, 37)_ = 1.34, *p* = 0.25] showed no significant difference.

#### 3.1.2. Audiovisual Modulation

To compare musicians and non-musicians when visual cues predict the upcoming sound in AV speech perception, a two-way ANOVA was conducted to examine the effect of the condition (AO vs. AV−VO) and participants' background (musicians vs. non-musicians) on N1 and P2 amplitudes and latencies. Results from the main effect of condition for N1 amplitude [*F*_(1, 37)_ = 3.94, *p* = 0.05] N1 latency [*F*_(1, 37)_ = 24.46, *p* = 0.00001], P2 amplitude [*F*_(1, 37)_ = 18.00, *p* = 0.0001] and P2 latency [*F*_(1, 37)_ = 3.95, *p* = 0.05] consistently showed lower latencies and amplitudes in AV−VO compared to the AV condition (Table 4).

**Table 4:** Summary of F-statistics of main effects and interactions.

	**Event-related potential (ERP)**				**Inter-trial phase coherence (ITPC)**			
	**N1**		**P2**					
	**Amplitude**	**Latency**	**Amplitude**	**Latency**	**Delta**	**Theta**	**Alpha**	**Beta**
Condition (AO vs. AV−VO)	3.94^*^	24.46^***^	18^***^	3.95^*^	6.12^*^	5.75^*^	7.25^*^	5.46^*^
Background (musicians vs. non-musicians)	0.08	3.26	2.04	0.21	0.32	0.57	10.58^**^	1.99
condition × background	4.99^*^	0.039	0.19	1.66	0.46	0	4.65^*^	2.23

* *p ≤ 0.05*,

** *p < 0.001*,

*** *p < 0.0001*.

As summarized in Table 4, results also showed a statistically significant interaction between condition and background for N1 amplitude [*F*_(1, 37)_ = 4.99, *p* = 0.032]. Following that, a *post-hoc* comparison using a paired-sample *t*-test for N1 amplitude showed that for musicians the N1 amplitude for AV−VO was significantly lower than for the AO condition, [*t*_(17)_ = −3.72, *p* < 0.001; Bonferroni corrected]. However, no corresponding difference was observed for non-musicians [*t*_(20)_ = 0.15, *p* = 0.87] (Table 3). As illustrated by the shift function in Figure 2B, musicians and non-musicians showed different patterns for N1 amplitude in AV−VO compared to the AO condition.

Results for the two-way ANOVA on N1 latency [*F*_(1, 37)_ = 0.03, *p* = 0.84], P2 amplitude [*F*_(1, 37)_ = 0.19, *p* = 0.66] and P2 latency [*F*_(1, 37)_ = 1.66, *p* = 0.2] showed no significant interaction between condition (AO vs. AV−VO) and background (musicians vs. non-musicians).

To investigate if the delayed AO N1 latency in musicians contributes to the N1 suppression effect in the AV−VO condition, a Pearson correlation coefficient was computed and showed no significant correlation between AO N1 latencies and AV−VO N1 amplitudes for musicians [*r* = −0.029, *n* = 18, *p* = 0.9].

**Figure 3:**
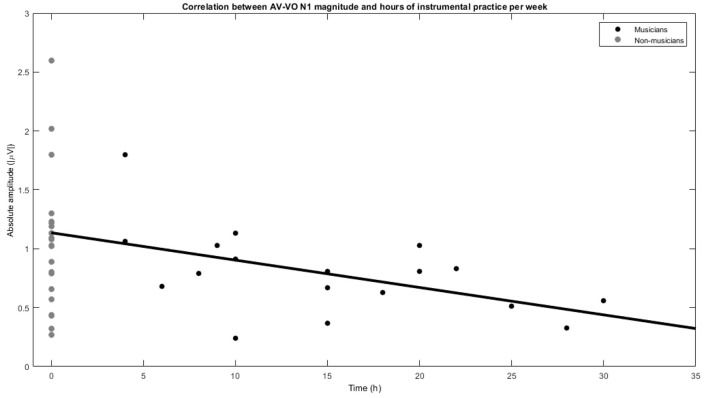
Correlation between N1 magnitude in AV−VO and hours of instrumental practice per week for musicians and non-musicians.

Musicians showed a lower AV−VO N1 amplitude than non-musicians, and to further investigate the relation between the effect of musical experience on the magnitude of AV−VO N1 amplitude and background information of the musicians, two-tailed Pearson correlation coefficients were computed. No significant correlation was observed between AV−VO N1 amplitude for musicians and either age of starting a musical instrument [*r* = 0.32, *n* = 18, *p* = 0.18] or years of musical training [*r* = −0.31, *n* = 18, *p* = 0.20]. However, as illustrated in Figure 3, a significant negative correlation was found between hours of practice per week and AV−VO N1 amplitude magnitude for musicians [*r* = −0.51, *n* = 18, *p* = 0.02].

**Figure 4:**
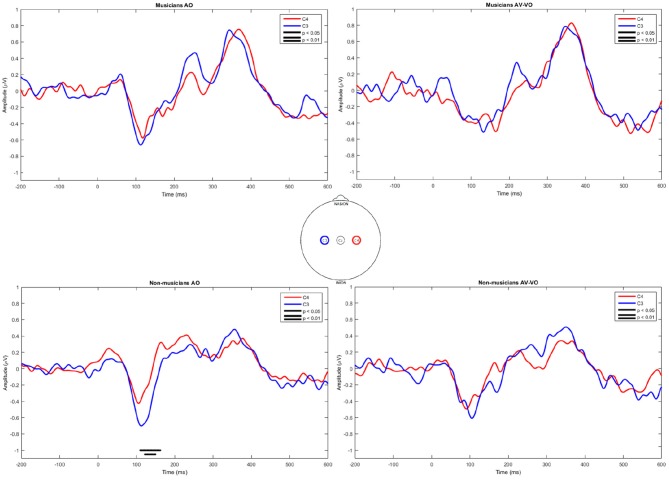
Point-by-point two-tailed *t*-test for event-related potentials in AO and AV−VO at C3 and C4 for musicians and non-musicians.

Finally, C3 and C4 for musicians and non-musicians were inspected lateralization and results are shown in Figure 4. In the AO condition musicians showed no significant difference between C3 and C4, whereas non-musicians showed higher amplitudes at C3 between 110 ms and 162 ms. For the AV−VO condition neither group showed a significant laterality.

### 3.2. Inter-trial Phase Coherence (ITPC)

**Figure 5:**
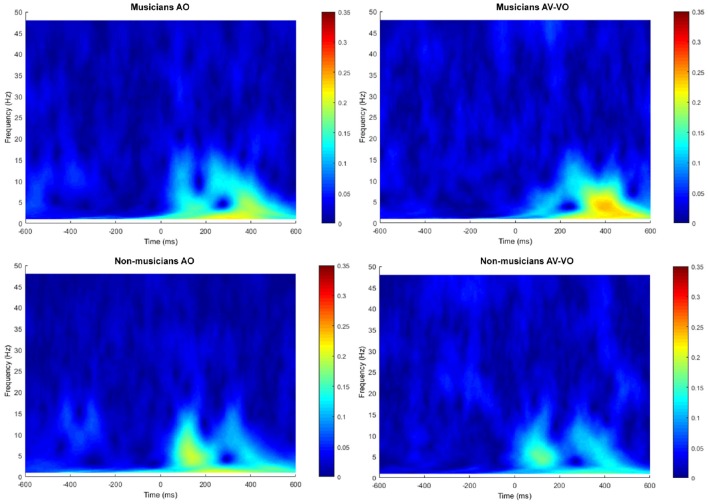
Trial-to-trial phased-locking measured by ITPC for audio only (AO) and audiovisual minus video only (AV−VO).

Figure 5 shows the trial-by-trial phase spectrum at Cz for AO and AV−VO both for musicians and non-musicians.

Separately for each of the frequency bands, a two-way ANOVA was conducted to examine effects of the condition (AO vs. AV−VO) and its interaction with participants' background (musicians vs. non-musicians). Results from the main effect of condition (AO vs. AV−VO) were significant for delta [*F*_(1, 37)_ = 6.12, *p* = 0.01], theta [*F*_(1, 37)_ = 5.75, *p* = 0.02], alpha [*F*_(1, 37)_ = 7.25, *p* = 0.01], and beta [*F*_(1, 37)_ = 5.46, *p* = 0.02] (Table 4). These results indicated that ITPC was significantly lower in AV−VO in comparison to AO across groups (Table 3).

The interaction between condition (AO vs. AV−VO) and background (musicians vs. non-musicians) was significant for trial-by-trial phase locking alpha activity [*F*_(1, 37)_ = 4.65, *p* = 0.03]. A *post-hoc* comparison using the paired-sample *t*-test for ITPC in alpha-band activity showed that alpha activity for musicians was significantly lower in the AV−VO condition relative to the AO condition [*t*_(17)_ = 2.46, *p* < 0.025; Bonferroni corrected], no corresponding difference was observed for non-musicians [*t*_(20)_ = −0.53, *p* = 0.60].

Results from the two-way ANOVA for delta [*F*_(1, 37)_ = 0.46, *p* = 0.5], theta [*F*_(1, 37)_ = 0.009, *p* = 0.92], and beta [*F*_(1, 37)_ = 2.23, *p* = 0.14], showed no significant interaction between condition (AO vs. AV−VO) and the background (musicians vs. non-musicians) of the participants.

To investigate if the N1 suppression in AV−VO correlates with alpha suppression in AV−VO in musicians, a Pearson correlation coefficient was computed and showed no correlation between AV−VO N1 amplitudes and AV−VO alpha ITPC in musicians [*r* = 0.07, *n* = 18, *p* = 0.77].

In summary, results for auditory speech perception showed that even though musicians had an N1 which on average was slightly later than for non-musicians, the groups did not significantly differ. Moreover, comparing auditory and AV speech perception, both groups showed lower N1 latency and P2 amplitude and latency as the results of the visual cues predicting the upcoming sound in AV speech perception. Musicians also showed lower N1 amplitude in AV perception. Delta, theta, and beta were also lower in AV perception compared to auditory perception for both groups, but musicians also showed lower alpha in AV speech perception, while non-musicians did not show this pattern.

## 4. Discussion

The current study first aimed to examine the effect of musical experience on auditory speech perception by comparing musicians and non-musicians based on their N1 and P2 amplitudes and latencies. The results showed that although musicians had an N1 which on average was slightly later than for non-musicians, the groups did not significantly differ. Moreover, as previous meta-analysis research (Baart, 2016), where musical experience varied, showed that AV modulation does not always lead to N1 amplitude reduction, the current study extends previous research by investigating whether AV modulation of speech is modified by previous AV experiences, such as playing a musical instrument. In AV speech perception, both groups showed lower N1 latency and P2 amplitude and latency as the results of the visual cues predicting the upcoming sound in AV speech perception. Musicians also showed lower N1 amplitude in AV perception compared to auditory perception, while non-musicians did not show this pattern at N1. A novel contribution of the current study is its use of ITPC analysis to examine the effect of musical experience in auditory and AV speech perception. Results showed that ITPC in delta, theta, and beta were also lower in AV perception compared to auditory perception for both groups. However, musicians, compared to non-musicians, showed lower alpha in AV speech perception.

### 4.1. Auditory Speech Perception

Previous studies on audio speech perception found variation in the results for N1 between musicians and non-musicians (e.g., Musacchia et al., 2008; Ott et al., 2011; Kühnis et al., 2014; Meha-Bettison et al., 2018). This variation in N1 is not limited to the direction of N1 amplitude and latencies; while some studies showed a difference for N1 between musicians and non-musicians (Ott et al., 2011, voiced and unvoiced stimuli; Meha-Bettison et al., 2018, syllable perception in noise) others observed no group difference at N1 in response to auditory stimuli, but an enhancement in P2, for musicians compared with non-musicians (for speech stimuli, Bidelman et al., 2014; for music stimuli, Kuriki et al., 2006; Baumann et al., 2008). Current findings showed no significant difference between musicians and non-musicians at N1 in auditory speech perception. However, although not statistically reliable, musicians were inclined to have a delayed N1 latency in comparison to their non-musician counterparts. In contrast to the current findings, Musacchia et al. (2008) found an increased N1 amplitude and, a reduced latency for musicians relative to non-musicians. Notably, they also had a different auditory stimulus (synthesized /da/ syllable with a male voice) presented with a captioned video and used a counting task. A plausible explanation for the different results for N1 latency in the auditory condition could be that the captioned auditory stimuli in comparison to auditory only syllable might lead the participants to predict the auditory signal based on the visual cues (captions) (Lange, 2013). Furthermore, musicians might predict the auditory signal based on the captions faster compared to non-musicians, that leads to a reduction for N1 latency. Therefore, reduced N1 latency might be a result of faster predicting the auditory signal rather than just the auditory perception. Another study by Kühnis et al. (2014) found a lower N1 amplitude for musicians in a passive listening task with synthetic vowel stimuli. Together these findings indicate that the difference between musicians and non-musicians show variation for N1 amplitudes and latencies.

Similar to the N1 component, P2 modulation with musical background also shows variation across studies. For example, some studies (e.g., Bidelman et al., 2014) have shown musicians to have enhanced P2 amplitude compared to non-musicians. On the contrary, and in line with the current study results, others (Baumann et al., 2008; Musacchia et al., 2008; Kühnis et al., 2014) found no significant difference between musicians and non-musicians in auditory perception for P2 amplitude and latency.

Such variation in N1 and P2 findings for musicians and non-musicians leads some studies to conclude that both N1 and P2 are prone to plasticity effects of musical experience (e.g., Shahin et al., 2003, 2005; Musacchia et al., 2008) and should be considered an N1-P2 complex rather than attributing separate roles to N1 and P2 for musical experience (Baumann et al., 2008). However, these variations in the musicians' N1 and P2 results across studies may also be driven by factors such as the experimental task, since N1 reflects the basic encoding of acoustic information (Näätänen and Picton, 1987; Näätänen et al., 2011) and is prone to inter-individual variability (Liem et al., 2012; Tan et al., 2016) but also is sensitive to attention (Lange, 2013).

Left laterality is related to right-handedness (e.g., Beaton, 1997) and response to segmental speech materials (e.g., Molfese et al., 1975; Zatorre et al., 1992; Tervaniemi and Hugdahl, 2003). Non-musicians have also been shown to have greater leftward asymmetry than musicians. In the current study all participants were right-handed and responded to speech stimuli, and finding greater left laterality for non-musicians than musicians was therefore expected. Furthermore, the musicians all had piano or keyboard as their main or secondary instrument, implying bimanual activity while playing the instrument, and consistent with indications for greater bilateral activity playing using both hands such as a piano (Haslinger et al., 2004; d'Anselmo et al., 2015), no laterality was observed for the musicians in the current study.

### 4.2. Audiovisual Modulation in Speech Perception

#### 4.2.1. Event-Related Potentials (ERPs)

Previous research (Baart, 2016) suggested that phonetically congruent visual cues predicting an upcoming speech sound modulate AV speech perception and lead to lower N1 and P2 amplitudes and latencies. In the current study, for both groups, N1 and P2 amplitudes and latencies for auditory speech were compared with AV speech in an additive model to examine if N1 and P2 amplitudes and latencies are lower in AV speech perception compared to audio speech perception. Musicians and non-musicians both showed bilateral activity in AV perception as well as similar AV modulation, with lower N1 latency, and lower P2 amplitude and latency in AV speech compared to auditory speech. The AV modulation effect for N1 latency in both groups is in line with previous research on AV modulation in speech (Stekelenburg and Vroomen, 2007; Paris et al., 2017), showing that visual cues predicting the upcoming sound reduce N1 latency. As is also found in the current study, having visual speech together with congruent audio speech also decreases P2 amplitude and latency (Van Wassenhove et al., 2005; Arnal et al., 2009).

Notably, the N1 amplitude suppression found in the current study was not observed in previous research, nor was the experimental design directly comparable. Musacchia et al. (2008) compared musicians and non-musicians separately for auditory and AV speech without controlling the predictive effect of visual cues, and they observed a higher N1 amplitude for musicians compared to non-musicians in AV speech. The current study evaluated AV modulation due to visual prediction of an upcoming sound in AV speech perception by directly comparing the auditory and AV−VO for each group. With this approach, findings for musicians showed a significantly lower N1 amplitude in AV speech compared to auditory speech perception, while non-musicians did not display such a deflection.

From this perspective, the current findings for N1 amplitude suppression for musicians are consistent with other findings for AV perception. Paris et al. (2016a, 2017) demonstrated that AV modulation at N1 in response to recently learned non-speech stimuli (figures and sound) depends on the visual cues predicting when the upcoming sound is coming and what is coming. They further suggested that the ecological stimuli used in Stekelenburg and Vroomen (2007) showed such AV modulation at N1, with the prediction of regularities learned over the life span. The current study takes this a step further addressing what happens when perceivers have a more precise temporal prediction due to previous musical experience, and in particular, whether this experience increases sensitivity of visual cues for predicting the upcoming sound in AV speech perception. Previous research showed subtle differences between musicians and non-musicians in AV perception (Musacchia et al., 2008; Lee and Noppeney, 2011; Paraskevopoulos et al., 2012; Proverbio et al., 2016). For example, a perceiver's prior expectations can influence temporal integration, and relevant training can generate more precise temporal predictions (Noppeney and Lee, 2018) leading to higher sensitivity to AV misalignments (for behavioral study see, Behne et al., 2013, for EEG study see, Behne et al., 2017). Another study (Petrini et al., 2009a) has shown that while musicians have a more refined integration window for AV music perception compared to non-musicians, they are also more accurate at predicting an upcoming sound when the visual information is missing in AV music perception (Petrini et al., 2009b). An fMRI study (Petrini et al., 2011) has shown the difference in lateralization of brain activity between musicians and non-musicians during a simultaneity judgment task. Musicians' brain activation was reduced bilaterally in the cerebellum, and the left parahippocampal gyrus. These studies suggest that as musicians have more refined integration windows to combine and more precisely predict visual and auditory information, they might show a decreased response as AV information is combined more effectively over time (Petrini et al., 2009b; Costa-Faidella et al., 2011; Lee and Noppeney, 2014; Lu et al., 2014; Bidelman, 2016). In other words, the decreased amplitude at N1 may be a consequence of musical expertise in temporal bindings.

These studies suggest that musicians more accurately predict the temporal relationship between the audio and visual signals, reducing the uncertainty about the temporal occurrence of the audio signal and thereby leading to a lower N1 amplitude (Costa-Faidella et al., 2011).

As mentioned earlier, for auditory speech perception, that N1 was on average 5 ms later for musicians than non-musicians did not significantly differ. This raises two questions: First, do musicians actually have more AV modulation for N1 latency compared to non-musicians, and in particular, does AV modulation for N1 latency depend on the latency magnitudes for the audio speech? The current findings for musicians do not show more AV modulation for N1 latency compared to non-musicians. In other words, musicians did not show more N1 suppression due to the visual cues predicting the upcoming sound, suggesting that there might be a ceiling effect for AV modulation for N1 latency reduction.

A further issue is the possibility that a later N1 for auditory speech may carry over as N1 amplitude facilitation in audiovisual speech perception. As Arnal et al. (2009) suggested, there is a pathway from the visual cortex through STS, which is sensitive to the congruency of the stimuli and occurs approximately 20 ms after N1. Therefore, if musicians have delayed N1 latency in audio speech which can be modified by the later pathway from the visual cortex through STS, musicians' AV modulation for N1 amplitude might be modified by the feedback loop from STS. Findings from the current study showed no correlation between N1 latency in audio speech and AV modulation for N1 amplitude for musicians, implying that if N1 for musicians would be slightly delayed in audio speech perception, it did not modify their AV modulation for N1 amplitude in AV speech perception by the later pathway through STS.

Musicians, compared to non-musicians, showed a lower N1 amplitude as a consequence of musical experience in AV perception. To clarify whether the background information on musicians' previous musical experience contributes to their N1 amplitude magnitude in AV perception, the correlation between the N1 amplitude magnitude in AV perception and age of starting a musical instrument, years of musical practice, and hours of playing an instrument per week were examined for musicians. Results showed no significant correlation between the magnitude of N1 amplitude in AV perception and the age of starting a musical instrument or years of musical practice. However, a significant negative correlation was observed between musicians' N1 amplitude magnitude in AV perception and hours of instrumental practice per week, indicating that as musicians increase time practicing weekly, they show a lower magnitude N1 amplitude when visual cues predict an upcoming sound in AV perception. The current findings are in line with Lee and Noppeney (2014), suggesting weekly musical practice as an indicator of the effect of musical experience in AV speech perception. These results imply that actively practicing a musical instrument is related to N1 amplitude magnitude in AV perception through previous musical experience.

#### 4.2.2. Inter-trial Phase Coherence (ITPC)

N1 and P2 components have been correlated with ITPC in lower frequencies (<30 Hz) (Edwards et al., 2009; Koerner and Zhang, 2015; van Diepen and Mazaheri, 2018) and ITPC in these lower frequency bands have been shown to play an essential role in AV speech perception (Arnal et al., 2011; Arnal and Giraud, 2012). For example, Arnal et al. (2011) showed that early cortical auditory evoked potentials are correlated with theta band activity, which has been correlated to the intelligibility of speech (Luo and Poeppel, 2007). Consistent with previous findings, both groups in the current study showed lower delta, theta and, beta-band activity in AV speech relative to audio speech perception. The groups did not differ in their lower ITPC values for delta, theta, and beta activity in AV speech perception. Other recent findings suggested that theta activity is suppressed in response to AV speech perception (Arnal et al., 2011; Lange et al., 2013). Theta activity has been related to mouth movements by a talker during speech production (Chandrasekaran et al., 2009) and, together with delta activity, reflects visual predictiveness of the stimuli (Arnal and Giraud, 2012). The predictiveness of the visual cues modifies the phase in delta-theta oscillation, which can contribute to explaining the cross-modal benefits of visual speech. Delta and theta-band activities also signal, in a feedforward loop, the processing of correctly anticipated stimuli. Previous research has mostly focused on later (i.e., relative to the current study) AV modulation of delta activity (350-550 ms) which is related to the post-sensory speech processes (Arnal et al., 2011), whereas in line with the current study, Stefanics et al. (2010) suggested that delta activity first decreased and then increased in response to correctly predicted stimuli. Furthermore, beta activity, together with the delta band, predicts the temporal accuracy of the upcoming stimuli (Arnal et al., 2015). Beta band activity, in line with the current study, is lower in response to phonologically congruent AV stimuli (Arnal et al., 2011). This indicates that beta activity is related to the prediction errors and feedback loops (Arnal et al., 2011; Arnal, 2012; Arnal and Giraud, 2012), and increases in response to incongruent AV stimuli (Arnal et al., 2011) and omission of an expected sound (Fujioka et al., 2009).

However, in the current findings for ITPC in the alpha band showed a significant interaction between musical background and condition, indicating that even though both groups showed alpha desynchronization in response to AV speech in comparison to the audio speech, musicians showed more alpha desynchronization than non-musicians. Corresponding patterns were not observed for delta, theta, and beta oscillations. Suppression of ITPC in the alpha band in both groups due to visual cues to the upcoming audio signal in the AV condition is in line with studies on anticipatory attention with speech (Arnal and Giraud, 2012; Gisladottir et al., 2018), non-speech (Bastiaansen and Brunia, 2001; Bastiaansen et al., 2001) and tactile stimuli (van Ede et al., 2014). When visual cues are predicting the upcoming stimuli, the visual stimulus onset itself leads to substantial decreases in the amplitude of ongoing alpha oscillations (Foxe and Snyder, 2011; Arnal and Giraud, 2012). Despite different experimental paradigms and different stimuli, these studies illustrated that attention, modulated by alpha oscillation orientates toward the upcoming stimuli to facilitate perception. This is similar to AV modulation in AV speech studies showing that congruent visual cues coming before the audio signal starts, modulate audio perception by predicting the upcoming sound. In the current study, musicians showed more alpha desynchronization than non-musicians, which is consistent with previous research, observing different alpha modulation in musicians in speech (Kühnis et al., 2014) and music tasks (Overman et al., 2003) compared to non-musicians. The current results indicating that when the visual cues predict the upcoming sound, musicians compared to non-musicians had more alpha desynchronization in AV speech as a result of focusing their attention to the visual cues compared to auditory speech perception.

Alpha oscillation, which is the most dominant signal measurable in human M/EEG (Strauß et al., 2014), might not be a unitary response but indeed functionally dissociated. Alpha oscillation is the only known frequency domain that responds to a stimulus or task demand with either a decrease or increase in frequency power, which might occur early in the primary auditory cortex (Strauß et al., 2014). Generally, brain regions which are activated during a task exhibit desynchronization, whereas regions associated with irrelevant or interfering tasks exhibit an increase in alpha oscillation (Klimesch, 2012). An alternative theory is that alpha-band activity is not really increased in areas processing irrelevant or interfering tasks, but rather reflects a return to the baseline level, while maintenance of relative alpha desynchronization in areas processing potential target information reflects preparatory enhancement (Foxe and Snyder, 2011). In either case, the modulation of the alpha activity is not only a bottom-up process but depends on top-down attentional control (Buffalo et al., 2011; Strauß et al., 2014). The alpha-band mechanism for gating attention has been observed across a variety of tasks for anticipation in multisensory modalities (Foxe and Snyder, 2011). The prevalence of studies suggesting alpha-band oscillation mediating attentional gating implies that alpha-band activity may be a general mechanism for attentional gating of cortical processing (Foxe and Snyder, 2011).

In addition to attentional modulation, alpha-band activity has also been studied in speech perception. For example, while processing vowels, alpha activity regulates temporal realignment of phase (Bonte et al., 2009) and can reflect a training-related tuning of bilateral auditory-related brain regions during speech processing (Bonte et al., 2009). Alpha activity can be an indicator of cognitive load (Luo et al., 2005), and word integration (Wilsch et al., 2014), and is also known as an active inhibitory mechanism which gates sensory information processing (Arnal and Giraud, 2012). Also, in comparison with oscillatory responses to AV congruent and incongruent stimuli, alpha power is higher in response to congruent AV speech relative to incongruent AV speech (Paris et al., 2016b). These findings imply that the role of alpha activity in speech perception is in line with the general observation that alpha activity shows desynchronization related to processing relevant information (Arnal and Giraud, 2012).

### 4.3. Relationship Between Event-Related Potentials (ERPs) and Inter-trial Phase Coherence (ITPC)

In the current study, findings showed no correlation between lower N1 amplitude and alpha desynchronization in response to AV speech for musicians. The lack of correlation between N1 amplitude and alpha oscillation may seem slightly puzzling since according to previous literature (e.g., Edwards et al., 2009), oscillatory activities with a frequency range between 4 and 15 Hz significantly correlated with ERP components. However, the current findings are consistent with Kühnis et al. (2014), who also observed no correlation between musicians' N1 amplitude and alpha-band activity. Their results did however, showed a correlation between beta-band activity and N1 amplitude. Furthermore, as formula 1 shows, ITPC reflects the contribution of signal components at each latency and frequency to the amplitude of ERP components, such as N1. Therefore, despite the effect of phase desynchronization of alpha ITPC on N1 amplitude, ITPC in higher frequency bands, especially theta and delta, can lead to the lack of correlation between N1 amplitude and ITPC in the alpha-band for musicians in AV speech perception.

### 4.4. Attention

As has been discussed, attention can modify the neural correlation of multisensory modulation (Talsma et al., 2010; Lange, 2013; Paris et al., 2016a) and speculation over the extent to which the present N1 amplitude results are modulated by the effects of attention on multisensory processing is inevitable. Based on the current findings, musicians showed lower N1 amplitude in response to AV speech compared to auditory speech, while non-musicians do not show this suppression. This is consistent with previous research on AV modulation in speech perception suggesting that the amplitude of N1, which was defined between 50 and 95 ms, is enhanced for attended sound and lower for an upcoming audio signal predicted by visual cues (Lange, 2013; Paris et al., 2016a), where attention enhancement at N1 only occurs when the upcoming sound is unpredicted (Paris et al., 2016a).

A second point related to attention is that findings from the current experiment are based on the non-target trials for which participants passively watched the stimuli without responding and might not have selectively attended those trials. That possibility remains, despite a chinrest being used to ensure they were indeed watching the stimuli, and they were responding to random target trials to ensure that they were attending to the whole experiment. However, previous research on musicians‘ auditory perception has shown that musical expertise enhancing N1 amplitude might not be sensitive to selective attention (Hillyard et al., 1973; Baumann et al., 2008) and that attention has a different time course (between 150 and 200 ms after sound onset) than the influence that musical expertise has on N1 amplitude (Baumann et al., 2008). These studies, together with the current findings, indicate that although attention may influence N1 amplitude, the effect of musical training cannot be reduced to an attention effect (Besson et al., 2011).

Third, as seen in Figure 2A between 300 and 400 ms after sound onset, a late positivity peak occurs with time range and polarity features similar to the P300 component. P300 is usually elicited by an “oddball” paradigm and is sensitive to the attentional resources that are involved during the task (Polich, 2007). However, the paradigm in the current experiment differs from an “oddball” paradigm since, here, trials that did not have targets were analyzed. Considering the experimental paradigm in the current study, this positive peak may plausibly be explained as an off-set response following the P1-N1-P2 complex to the speech stimuli (Alain and Tremblay, 2007; Han, 2010), and thereby not directly related to attention.

### 4.5. Consideration for Musical Experience

An essential difference between prior studies (e.g., those included in Baart, 2016) and the current one is controlling the previous musical background of the participants. For this study, the musician group consists of expert instrumentalists for their age group, specifically with no dancing or singing experience. Musicians with singing and dancing training were excluded from this study since this training might lead to structural and functional differences compared to the instrumental training, and thereby may influence audio perception (Halwani et al., 2011; Poikonen et al., 2018). For example, vocalists, in comparison to instrumentalists have been shown to have structural differences in their arcuate fasciculus, a projector tract that connects the STS and frontal regions (Halwani et al., 2011). Dancers have also shown structural differences in the arcuate fasciculus, sensory-motor, pre-motor cortex, and STS compared to non-dancers (Hänggi et al., 2010). In addition, dancers have an optimal auditory and somatosensory connection for synchronizing the execution of movements with the auditory rhythm (Brown et al., 2005). These studies support the idea of distinguishing the different musical experiences involved in AV speech perception and predictive coding since they might have a confounding effect on brain areas, such as the auditory cortex, motor cortex, and STS (Arnal et al., 2009; Arnal, 2012). In further research, on the influence of different forms of AV experience, such as dancing and singing, may further contribute to our understanding of differences in the use of visual cues in predicting the upcoming sound in AV speech perception.

## 5. Conclusions

In the current study, first, to replicate previous investigations with auditory speech, musicians and non-musicians were compared based on their N1 and P2 amplitudes and latencies evoked by the auditory syllable /ba/. Results showed that for auditory speech musicians were inclined to have a delayed N1 latency, albeit not significantly different from their non-musician counterparts. Musicians' N1 latency did not contribute to their results from AV speech perception. Furthermore, the current study supports previous research on AV modulation at N1 and P2, suggesting that the phonetically congruent visual cues predicting the upcoming sound lead to lower N1 latency and P2 amplitude and latency. Likewise, that ITPC in the delta, theta, and beta bands were lower in AV speech compared to the auditory speech perception is consistent with previous studies suggesting that early ERP components, such as N1 and P2, are correlated with ITPCs in low-frequency bands (<30 Hz).

The current study contributes to previous findings on multimodal perception by investigating whether the AV modulation of speech is modified by previous AV experience, such as musical training, and whether the musical background of the participants can explain some variation across previous studies (Baart, 2016). Findings suggest that previous musical experience modifies AV modulation due to the visual cues predicting the upcoming sound at N1 amplitude; while musicians showed N1 suppression due to AV modulation, non-musicians did not. While N1 amplitude is sensitive to the opposing influence of prediction and attention, lower N1 amplitude in AV speech perception shows the contribution of prediction for musicians. Moreover, musicians, in comparison to non-musicians, showed alpha desynchronization in AV speech compared to auditory speech perception, suggesting that they focus their attention on the visual cues which lead to predicting the upcoming sound. Together, current findings show that early sensory processing in AV speech perception can be modified by musical experience which, in turn, may explain some variation across previous studies.

## Data Availability Statement

The datasets generated for this study are available on request to the corresponding author.

## Ethics Statement

The studies involving human participants were reviewed and approved by Norwegian Centre for Research Data (NSD). The patients/participants provided their written informed consent to participate in this study. Written informed consent was obtained from the individual(s) for the publication of any potentially identifiable images or data included in this article.

## Author Contributions

Both authors contributed extensively to the work presented in this paper. MS and DB jointly conceived of the study and sketched the design. MS carried out the practical implementation of the project, carried out the EEG experiments and data analyses, and drafted the full paper. DB supervised all stages of the project. Both authors discussed the results and implications and contributed to the manuscript.

### Conflict of Interest

The authors declare that the research was conducted in the absence of any commercial or financial relationships that could be construed as a potential conflict of interest.
